# *De Novo* transcriptome characterization of *Dracaena cambodiana* and analysis of genes involved in flavonoid accumulation during formation of dragon’s blood

**DOI:** 10.1038/srep38315

**Published:** 2016-12-06

**Authors:** Jia-Hong Zhu, Tian-Jun Cao, Hao-Fu Dai, Hui-Liang Li, Dong Guo, Wen-Li Mei, Shi-Qing Peng

**Affiliations:** 1 Key Laboratory of Biology and Genetic Resources of Tropical Crops, Ministry of Agriculture, Institute of Tropical Bioscience and Biotechnology, Chinese Academy of Tropical Agricultural Sciences, No. 4 Xueyuan Road, Haikou 571101, China

## Abstract

Dragon’s blood is a red resin mainly extracted from *Dracaena* plants, and has been widely used as a traditional medicine in East and Southeast Asia. The major components of dragon’s blood are flavonoids. Owing to a lack of *Dracaena* plants genomic information, the flavonoids biosynthesis and regulation in *Dracaena* plants remain unknown. In this study, three cDNA libraries were constructed from the stems of *D. cambodiana* after injecting the inducer. Approximately 266.57 million raw sequencing reads were *de novo* assembled into 198,204 unigenes, of which 34,873 unique sequences were annotated in public protein databases. Many candidate genes involved in flavonoid accumulation were identified. Differential expression analysis identified 20 genes involved in flavonoid biosynthesis, 27 unigenes involved in flavonoid modification and 68 genes involved in flavonoid transport that were up-regulated in the stems of *D. cambodiana* after injecting the inducer, consistent with the accumulation of flavonoids. Furthermore, we have revealed the differential expression of transcripts encoding for transcription factors (MYB, bHLH and WD40) involved in flavonoid metabolism. These *de novo* transcriptome data sets provide insights on pathways and molecular regulation of flavonoid biosynthesis and transport, and improve our understanding of molecular mechanisms of dragon’s blood formation in *D. cambodiana*.

Dragon’s blood is a red resin excreted by a part of the genus *Dracaena* plants, which has been used as a famous traditional medicine since ancient times^1^. Modern pharmacological studies have found that this resinous medicine has anti-bacterial, anti-spasmodic, anti-inflammatory, analgesic, anti-diabetic, and anti-tumor activities, and is widely applied for the treatment of wounds, leucorrhea, fractures, diarrhea and piles as well as for intestinal and stomach ulcers^1^,^2^. In China, *Dracaena cochinchinensis* and *Dracaena cambodiana* were the mainly plant resources of dragon’s blood^3^. In nature, *Dracaena* plants grow very slowly, and only trees with 30–50-years-old have the possibility to produce a small amount of dragon’s blood. In addition, natural *Dracaena* resources have been destroyed severely owing to overexploitation^4^,^5^. Our previous studies found an effective inducer (authorized patent: ZL 201310207182.5) could induce the formation of the resin in the stem of *D. Cambodiana*^6^. However, the formation mechanism of dragon’s blood is still unknown. Phytochemical studies of dragon’s blood revealed that flavonoids are the main chemical constituents, while terpenes, steroids, saponins and phenols have also been identified as constituents^7^,^8^,^9^,^10^,^11^. Owing to a lack of *Dracaena* plants genomic information, the molecular basis of specific flavonoids biosynthesis and the regulation of formation in *Dracaena* plants remain unknown.

The flavonoids metabolism pathway (especially anthocyanin) has been well characterized in model plants at the genetic, biochemical and molecular levels^12^,^13^. Given the distribution of flavonoids vary differently among different plants, the molecular mechanisms of their biosynthesis, transport and regulation might be diverse and complex^14^. Therefore, it is essential to investigate the molecular mechanisms of flavonoids accumulation in *D. cambodiana.* However, the lack of the transcriptomic and genomic data has made it difficult to investigate the mechanisms of flavonoid accumulation and dragon’s blood formation in *D. cambodiana.*

Next generation sequencing platforms provide highly efficient tools to identify novel genes associated with biosynthesis of various secondary metabolites in non-model plant species^15^. Specially, it has been widely applied to investigate molecular mechanisms of flavonoids accumulation in plant species such as *Dioscorea alata*^16^, *Prunus avium*^17^, *Olea europaea*^18^ and *Gentiana straminea*^19^. Here, we present the transcriptome analysis of *D. cambodiana* using RNA-Seq complemented with HPLC flavonoid profiling to identify potential candidate genes involved in flavonoids accumulation. This study, therefore, may serve as a basis for the future discoveries on pathways and molecular regulation of flavonoid biosynthesis and transport in *D. cambodiana.*

## Results

### Increased flavonoids in the stem of *D. cambodiana* after injecting the inducer

**Figure 1:**
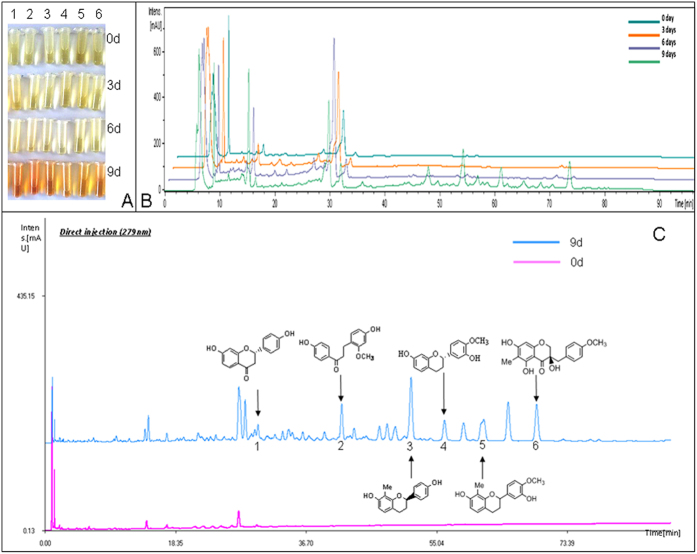
Changes of ethanol extracts from the stem of *D. cambodiana* after injecting the inducer. (**A**) The changes of color of ethanol extracts from the stem of *D. cambodiana* of 0 d, 3 d, 6 d and 9, d after injecting the inducer. (**B**) HPLC analysis of ethanol extracts from the stem of *D. cambodiana* of 0 d, 3 d, 6 d and 9, d after injecting the inducer. (**C**) Flavonoids were identified from ethanol extracts from the stem of *D. cambodiana* of 9, d after injecting the inducer.

The color of ethanol extracts from the stem of *D. cambodiana* changed from light yellow to brown after injecting the inducer (Fig. 1A). The compounds of ethanol extracts from the stem of *D. cambodiana* increased 9 d after injecting the inducer (Fig. 1B). We subsequently performed HPLC analysis to detect the flavonoids in the ethanol extract. As shown in Fig. 1C, some flavonoids were identified on the basis of their retention time compared to standards under the same condition (Table S1). The result shows the inducer can induce the formation of flavonoids in the stem of *D. cambodiana* after injecting the inducer.

### Assembling and annotation of *D. cambodiana* transcriptome

To elucidate the molecular mechanism and to find candidate genes of dragon’s blood formation, three cDNA libraries were obtained from the stems of *D. cambodiana* after injecting the inducer in 0 day, 3 days, 6 days respectively (designated 0d, 3d and 6d) and constructed. By sequencing on the platform of Illumina Hiseq 2000, total raw reads of 266.57 M were generated and trimmed to exclude low-quality reads (Table 1). To perform the *de novo* assembly, 217.5 M high-quality reads were further assembled into 198,204 unigenes with an average length of 495 bp, a maximum length of 21,883 bp and an N50 length of 562 bp. After removal of contaminant sequences, a total of 70,122 unigenes longer than 400 bps were selected for further analyses. The length distribution of the unigenes is illustrated in Fig. S1. The sequences ranging from 400 bp to 2,000 bp in length accounted for nearly 92.5% of the total. Up to 3,444 unigenes (4.9%) and 1,842 unigenes (2.6%) were 2, 000 bp to 3,000 bp and >3000 bp in length, respectively.

The unique sequences were annotated using BLASTx against the NCBI non-redundant nucleotide database (Nt), the Swiss-Prot protein database, the KEGG database, and the COG database. A total of 34,873 (17.5%) unique sequences could be matched to known genes in the public databases (Table S2). The proportion of the unigenes with BLASTx hits significantly increased for longer unigenes (Fig. S1). The BLASTx searches yielded hits for 18590 (28.7%) unigenes that were 400 bp to 2,000 bp, 3102 (90.1%) unigenes that were 2,000 bp to 3,000 bp, while 1748 (94.9%) of the unigenes longer than 3,000 bp were annotated. However, there were still a large number of unigenes (163,421, 82.5%) without BLASTX hits. Most of these sequences were short fragments, and some of them might be non-coding RNA sequences or new genes.

### Differentially expressed genes (DEGs) in the stem of *D. cambodiana* after injecting the inducer

**Figure 2:**
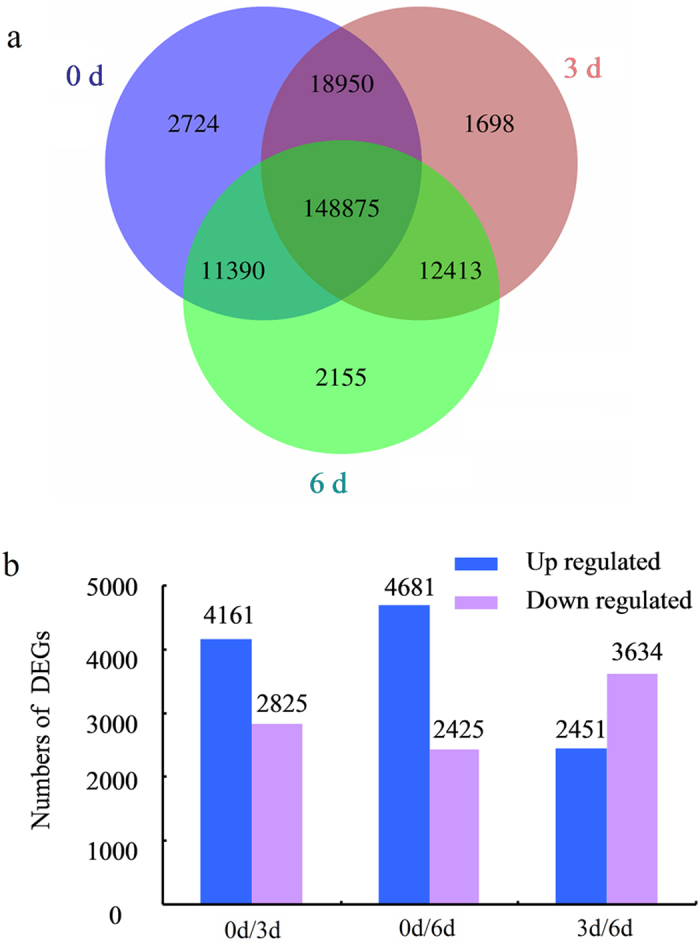
Analysis of global gene expression among three samples. (**a**) The number of common and unique unigenes expressed among three libraries. (**b**) The number of significantly (P-value ≤ 0.05 and at least two-fold change) up- and down-regulated transcripts between two compared samples.

Expression levels of unigenes were determined by aligning the RNA-Seq reads from each library to the assembly. As a result, a total of 148,875 genes were expressed in all three samples, and 2,724, 1,698, 2,155 unigenes were specifically expressed in 0d, 3d and 6d respectively (Fig. 2a). The differences in gene expression patterns were then analyzed for the pairs 0d and 3d, 0d and 6d, 3d and 6d based on the false discovery rate (FDR) ≤ 0.05, and fold change (FC) ≥ 1. A total of 6,986 genes were differentially expressed between the 0d and 3d libraries, among which 4161 were up-regulated and 2,825 were down-regulated. Between the 0d and 6d libraries, 7,106 genes were differentially expressed, including 4,681 up-regulated and 2,425 down-regulated genes. The 3d and 6d libraries showed 6,085 DEGs, including 2451 up-regulated and 3634 down-regulated (Fig. 2b).

### GO enrichment and KEGG pathway analysis of DEGs

**Figure 3:**
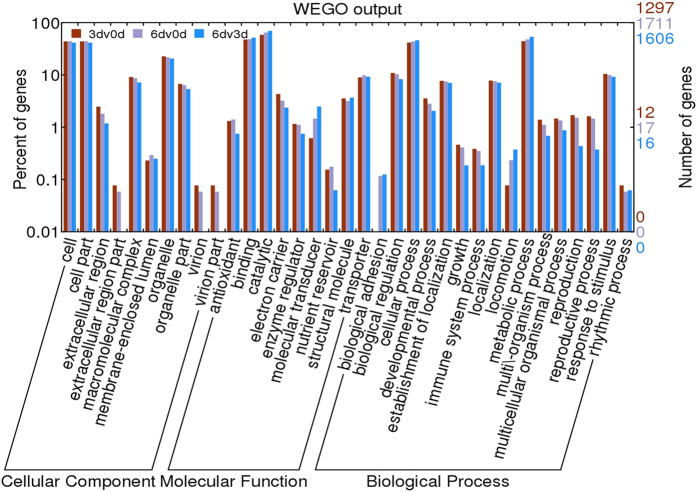
Functional classification of differentially expressed genes between each two of three group based on GO classification. Gene Ontology (GO) terms are summarized in three main categories of biological process, molecular function and cellular component.

We did functional categorize to the differential expression unigenes by using gene ontology (GO) terms. The GO terms allow for the definition and standardization of the properties of gene products in any organism. The 3d against 0d libraries, 1,297 different genes were annotated. Of the three main sub ontologies, molecular function was the highly represented, with 1,129 unigenes followed by biological process with 795 unigenes and finally, cellular component with 584 unigenes. Besides this, 1,711 and 1,606 unigenes were annotated in other groups (Fig. 3). GO enrichment analysis show detail information about the changes. GO enrich results with correct p-Value < 0.01 show “molecular function” and “cellular component” between treated and control group. The major sub-categories are list in Table S3, including electron carrier activity (GO: 0009055), extracellular region (GO: 0005576), nucleic acid binding transcription factor activity (GO: 0001071), transporter activity (GO: 0005215), membrane (GO: 0016020), membrane part (GO: 0044425). After further analysis, we found the genes involved in electron carrier activity (GO: 0009055) and extracellular region (GO: 0005576) are mostly the cytochrome P450s which are generally taking an active part in secondary metabolism.

The Kyoto Encyclopedia of Genes and Genomes (KEGG) pathways represent collections of manually drawn pathway maps and that are helpful for the understanding of the biological functions and interactions of genes. By mapping Enzyme Commission (EC) numbers to the reference canonical pathways, a total of 998, 1,296, 1,419 distinct genes matched the metabolic pathways in the KEGG pathway database at 3d/0d, 6d /0d, 6d /3d, respectively. Phenylpropanoid biosynthesis, flavonoid biosynthesis and flavone and flavonol biosynthesis were the most prominent classes (Table S4). From the KEGG result, we can find secondary metabolite biosynthesis was animate in the *D. cambodiana’s* transcriptome, where flavonoids, flavone and flavonols, isoquinoline alkaloids, stilbenoid, diarylheptanoid and gingerol biosynthesis were the most relevant pathways by KEGG enrichment (Table S4).

### Candidate genes involved in flavonoid biosynthesis

**Figure 4:**
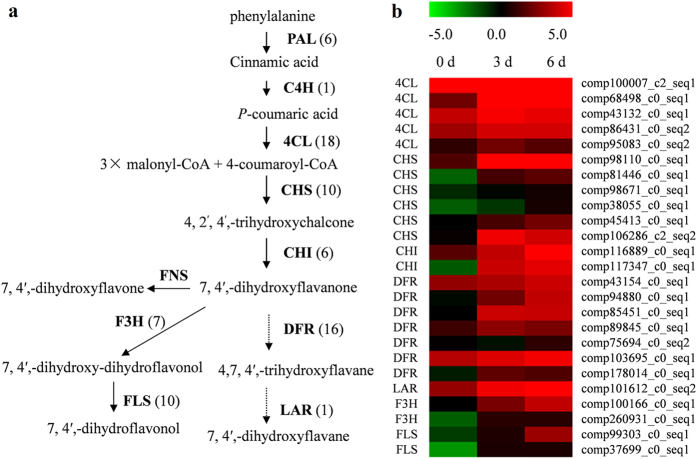
Putative flavonoids biosynthesis pathway. (**a**) Proposed pathway for flavonoids biosynthesis. The numbers in brackets following each gene name indicate the number of unigenes annotated to that gene. Enzyme abbreviations: PAL, phenylalanine ammonia lyase; C4H, cinnamate 4-hydroxylase; 4CL, 4-coumarate CoA ligase; CHS, chalcone synthase; CHI, chalcone isomerase; F3H, flavanone 3-hydroxylase; FLS, flavonol synthase; DFR, dihydroflavonol 4-reductase; LAR, leucoanthocyanidin reductase; FNS, flavone synthase. (**b**) Expression levels of the candidate unigenes coding key enzyme involved in flavonoids biosynthesis pathways. Green and red colors are used to represent low-to-high expression levels, and color scales correspond to the mean centered log_2_-transformed FPKM values.

The main components of dragon’s blood of *D. cambodiana* are flavonoids, especially flavanes, with 7,4′- dihydroxy flavane as basic skeleton. Up to now, the biosynthetic pathway of flavanes has not been reported in other plants. A brief schematic of flavonoid biosynthesis is shown in Fig. 4a, which is modified version from Lepiniec’s figure^20^ based on analyses of KEGG databases and chemical constituents from dragon’s blood of *D. cambodiana.* Flavonoids are synthesized via the phenylpropanoid pathway and are converted from phenylalanine to chalcone by the enzymes phenylalanine ammonia-lyase (EC 4.3.1.24, PAL, 6 unigene), cinnamate 4-hydroxylase (EC 1.14.13.11, C4H, 1 unigene), 4-coumarate CoA ligase (EC 6.2.1.12, 4CL, 18 unigenes) and chalcone synthase (EC 2.3.1.74, CHS, 10 unigene). Chalcone isomerase (EC 5.5.1.6, 6 unigene) catalyses the isomerisation of chalcones into flavanone. Flavanone can be converted either to flavonols through the subsequent action of flavanone 3-hydroxylase (EC 1.14.11.9, F3H, 7 unigenes) and flavonol synthase (EC 1.14.11.23, FLS, 10 unigenes) or to flavane through the action of dihydroflavonol 4-reductase (EC:1.1.1.219, DFR, 16 unigenes) and leucoanthocyanidin reductase (EC:1.17.1.3, LAR, 1 unigene). However, no unigene coding for flavone synthase (EC 1.14.11.22, FNS) was detected by RNA-sequencing. The reason may be that FNS genes were short fragments without sequence similarity.

It is known that most genes involved in flavonoid biosynthesis are coordinately expressed, consistent with flavonoid accumulation. We compared the differences in gene expression profile of those flavonoid biosynthesis genes to identify putative genes co-expressed with flavonoid accumulation. Among the above described genes involved in the flavonoid biosynthesis, five 4CL unigenes, six CHS unigenes, two CHI unigenes, seven DFR unigenes, one LAR unigenes, two F3H unigenes and two FLS unigenes were significantly up-regulated after injecting the inducer, consistent with flavonoid accumulation (Fig. 4b). Those genes co-expressed with flavonoid accumulation might play important roles in flavonoid biosynthesis and dragon’s blood formation.

The chemical diversity of flavonoids increases enormously by undergoing a variety of modification reactions such as hydroxylation, glycosylation, and/or methylation. Cytochrome P450 (CYP), UDP-glycosyltransferase (UGT) and O-methyltransferase (OMT) have been shown to play roles in the modification of flavonoids^21^. In our study, 11 CYP, 14 UGT and 2 OMT unigenes were found to be significantly up-regulated after injecting the inducer, which may be involved in the modification of flavonoids in *D. Cambodiana* (Table S5).

### Candidate genes involved in flavonoid transport

**Figure 5:**
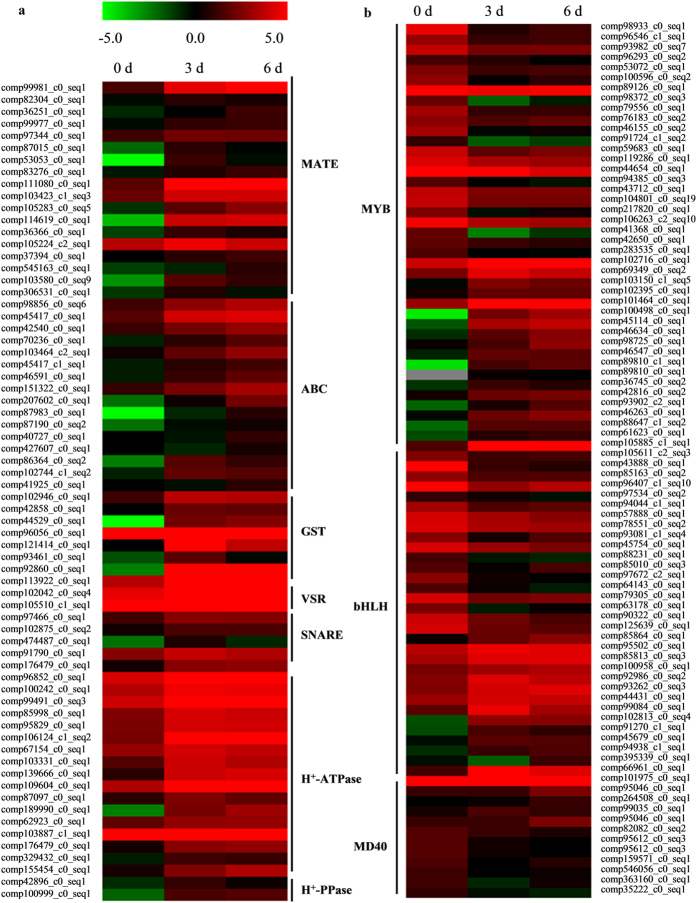
Expression levels of candidate unigenes involved flavonoids accumulation. (**a**) Expression levels of the candidate flavonoids transport-related unigenes up-regulated in stems of *D. cambodiana* after injecting the inducer. (**b**) Expression levels of TFs from DEGs involved in flavonoids accumulation. Green and red colors are used to represent low-to-high expression levels, and color scales correspond to the mean centered log_2_-transformed FPKM values.

Flavonoids are synthesized in the cytosol and transported into the vacuole for storage or to other destinations^22^,^23^. Multidrug and toxic compound extrusion protein (MATE) transporters, two subfamily of ATP-binding cassette (ABC) transporters (G-type (ABCG) and the multidrug resistance-associated protein (MRP)-type), glutathione S-transferases (GST), vacuolar sorting receptor (VSR) and soluble N-ethylmaleimidesensitive factor attachment protein receptors (SNARE), H^+^-ATPases and H^+^-PPases have been claimed to play roles in sequestration of flavonoids into the vacuole^22^,^23^. In *D. Cambodiana* transcriptome, 88 unigenes encoding MRP/ABCG, 34 unigenes encoding MATE, 20 unigenes encoding GST, 4 unigenes encoding VSR, 12 unigenes encoding SNARE, 74 unigenes encoding H^+^-ATPases and 13 unigenes encoding H^+^-PPases were found (Table S5). Transcripts expression analysis revealed 13 out of 35 unigenes encoding MRP were highly up-regulated after injecting the inducer; while only 3 out of 53 unigenes encoding ABCG were up-regulated (Fig. 5a). The results indicated that MRP-type ABC transporters might play important roles in flavonoid transport in *D. Cambodiana*. In addition, 18 MATE unigenes, 8 GST unigenes^24^, 2 VSR unigenes, 4 SNARE unigenes, 18 H^+^-ATPases and 2 H^+^-PPase unigenes were also found to be significantly up-regulated after injecting the inducer (Fig. 5a). These up-regulated unigenes might be involved in transportation of flavonoids from cytosolic synthesis to vacuolar accumulation in *D. Cambodiana*.

### Candidate transcription factors involved in flavonoid biosynthesis and transport

It is well known that transcription factors (TFs) play an essential role in regulating flavonoid biosynthesis and transport. In most species, flavonoid biosynthesis and transport is controlled by a ternary complex of MYB-bHLH-WD40, which generally regulate expression of many structural genes^25^. Based on our results, a total of 129, 82 and 93 unigenes were respectively predicted to code MYB, bHLH and WD40 proteins in the transcriptome database. Of these genes, the transcriptomic analysis detected 86 TFs that were differentially expressed in the stem of *D. cambodiana* after injecting the inducer, including 41 MYB unigenes (18 up-regulated and 23 down-regulated), 33 bHLH unigenes (15 up-regulated and 18 down-regulated), and 12 WD40 unigenes (5 up-regulated and 7 down-regulated) in the stem of *D. cambodiana* after injecting the inducer (Fig. 5b). These differentially expressed TFs might be involved in regulating flavonoid biosynthesis and transport in *D. cambodiana.*

### Real-time PCR validation of differential expression

**Figure 6:**
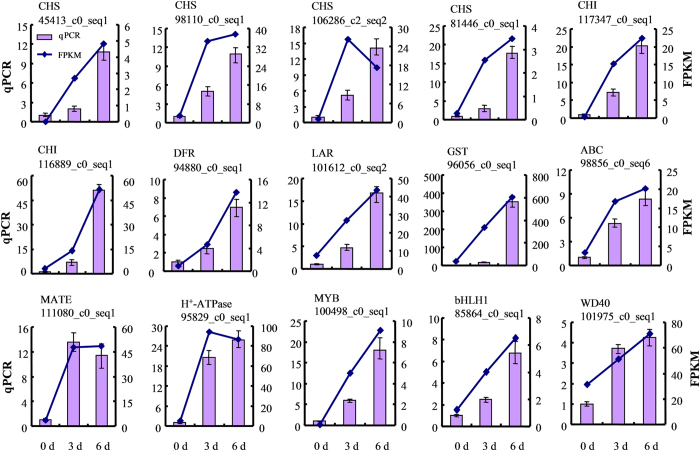
qPCR validations of 15 putative genes involved flavonoids accumulation. The histograms show the qPCR results of 15 unigenes involved in flavonoid biosynthesis, transport and regulation in stems of *D. cambodiana* after injecting the inducer in 0 d, 3 d, 6 d respectively; the line charts show the FPKM values of these unigenes. qPCR results represent the mean(±SD)of three biological replicates.

To confirm the unigenes obtained from sequencing and to further analyze the reliability of RNA-seq data in present study, 15 DEGs involved in flavonoid biosynthesis, transport and regulation were chosen for real-time quantitative PCR assay. The expression profiles of these unigenes are shown in Fig. 6. In general, the results showed that all the selected genes revealed similar expression pattern as observed in RNA-seq data. Therefore, our results provide reliable transcriptome and expression profile data for further investigations of key genes involved in flavonoid accumulation in *D. cambodiana.*

## Discussion

*D. cambodiana* is an important medicinal plant used as a source of dragon’s blood. The results of the analyses of chemical constituents indicate that the main components of dragon’s blood are flavonoids^7^,^8^,^9^,^10^,^11^. The limited transcriptomic data and genomic data hinder the study of the molecular mechanisms of flavonoids accumulation in *D. cambodiana.* In the present study, we firstly provideed the assembly transcriptome sequence for *D. cambodiana.* Based on the sequencing results, 198,204 unigenes were generated, 34,783 of which were similar to known proteins. All sequences involved in flavonoid biosynthesis, modification, transport and regulation were identified by searching these transcripts against sequence databases using the BLASTx search; and their expression levels were monitored by comparing them in the stems of *D. cambodiana* after injecting the inducer characterized by different flavonoid accumulation.

Flavonoids are synthesized by the phenylpropanoid metabolic pathway catalyzed by PAL, C4H and 4CL, which also serves as a starting point for the production of many other important compounds, such as coumarins, stilbenes, aurones and lignans^26^. In this study, six PAL, one C4H and eighteen 4CL unigenes were identified from the transcriptome data. There were no significant changes in the expression levels of PAL and C4H unigenes in stems after injecting the inducer, while five 4CL unigenes were significantly up-regulated. 4CL genes are divided into three classes, Class I, Class II and Class-4CL like^25^. It has been suggested that members of Class II are closely associated with flavonoid biosynthesis, those in Class I are involved in the biosynthesis of lignin and other phenylpropanoids, while the 4CL-like genes may be associated with other functions^27^,^28^,^29^. Among five 4CL DEGs, one is highly homologous to members of Class I and other four belong to Class-4CL like. In addition, all of the DEGs encoding key enzymes of flavonoid biosynthesis showed significantly up-regulated expression after injecting the inducer, such as CHS (6 unigenes), CHI (2 unigenes), DFR (7 unigenes), LAR (1 unigene), F3H (2 unigene) and FLS (2 unigene) (Fig. 5b), which were consistent with the accumulation of flavonoids in stems. These results indicated that the flavonoid biosynthesis in stems of *D. cambodiana* was activated obviously after injecting the inducer, while the upstream of which, phenylpropanoid metabolic pathway had no significantly change.

The transport of flavonoids from the cytosolic side of the endoplasmic reticulum (ER) to the vacuole may occur through three basic mechanisms: membrane transporter-, glutathione S-transferase (GST)-, or vesicle trafficking-mediated transport^22^,^23^. The proton gradient between the cytosol and the vacuole by H^+^-ATPases (and H^+^-PPases in the tonoplast) has been proposed to be the main driving force for the transport of some flavonoids^30^. ABCs and MATEs, as important transporters, have been claimed to play a role in sequestration of flavonoids into the vacuole by the membrane transporter-mediated transport system^31^,^32^,^33^,^34^,^35^. The vesicle trafficking-mediated transport involves flavonoid-containing vesicles releasing their content into the accumulation targets by fusion^36^, which requires VSR and SNARE proteins for budding, targeting, docking, fusion and recycling^23^. In *D. cambodiana* transcriptome, 18 MATE, 2 VSR, 4 SNARE, 18 H^+^-ATPase and 2 H^+^-PPase DEGs were found to be significantly up-regulated after injecting the inducer. Additionally, GSTs could act as flavonoid binding proteins, have been described as participating in vesicle uploading or vacuolar transpor^37^,^38^. We also found that eight out of 20 GST unigenes were up-regulated after injecting the inducer, and expression patterns of three GST DEGs were strongly consistent with dragon’s blood accumulation^24^. These results imply that the three distinct transport mechanisms (membrane transporter-, GST-, or vesicle trafficking-mediated transport) may be all present in *D. cambodiana*; transport of flavonoids may be a multifactorial process, involving different strategies and the contribution of several proteins. These findings provide insights into the research of flavonoid transport mechanisms in *D. cambodiana*. However, in recent years, significant progress in flavonoid transport mechanisms has been focused on anthocyanin, transport of other flavonoids such as flavanes has been limited. Further research is needed to confirm whether there are three similar transport mechanisms in *D. cambodiana* and which mechanism is prevalent in transport of flavanes.Controlled transcription of biosynthetic genes is one major mechanism regulating secondary metabolite production in plant cells^39^. MYB, bHLH and WD40 TFs are well known to be involved in flavonoid metabolism by regulating many structural genes, they can work individually or interact physically to form the MYB-bHLH-WD40 (MBW) complex^25^,^40^. Moreover, TFs also control the regulation of flavonoid transport. For example, AtTT2 regulates the expression of the MATE transporter gene *TT12* to control the flavonoids transport^41^, and the maize ABC transporter ZmMRP3 involved in anthocyanin transport is regulated by the R (bHLH family) and C1 (R2R3-MYB) TFs^35^; and anthocyanin-related glutathione S-transferase gene *LcGST4* was activated by LcMYB1, a key R2R3-MYB transcription factor that regulates anthocyanin biosynthesis in litchi^35^. Most of TFs are positive regulators in flavonoid biosynthesis, whereas a few of them have been identified as repressors in flavonoid pathway^42^. In our study, we first identified 42 MYB unigenes (19 up-regulated and 23 down-regulated) from the DEGs. Among these unigenes, one unigene (comp103150_c1_seq5) was highly homologous to *SbMYB2* in *Scutellaria baicalensis*, which was identified as regulators in the control of flavonoid biosynthesis^43^. Another one (comp102395_c0_seq1) was highly homologous to *MdMYB1*, which was also involved in anthocyanin biosynthesis and transport in apple^44^. In addition, 33 bHLH unigenes were also identified from the DEGs. Among these unigenes, comp100958_c0_seq1 was highly homologous to members of subfamily 2 of bHLH, which were identified as regulators in flavonoid or anthocyanin metabolism^45^. Comp85864_c0_seq1 was highly homologous to *GL3*, which has been reported to positively regulate anthocyanin synthesis^46^. The expression patterns of these 2 candidate bHLH genes were significantly up-regulated after injecting the inducer, consistent with flavonoid accumulation. Finally, 12 candidate WD40 unigenes were found among the DEGs, one of them (comp19899_c0_seq1) was expressed at highest abundance (with 10-fold or greater expression than other genes) in stems, the expression patterns of this gene was also positively correlated with flavonoid accumulation in stems. Although MYB, bHLH and WD40 TFs in regulating flavonoid metabolism have been well characterized in plants, the related TFs have not been reported in *D. cambodiana*. Further studies are still needed to determine whether the flavonoid metabolism in *D. cambodiana* is regulated by these candidate genes, and which is independently regulated by a single TF, or controlled by combinations of TF complexes.

**Figure 7:**
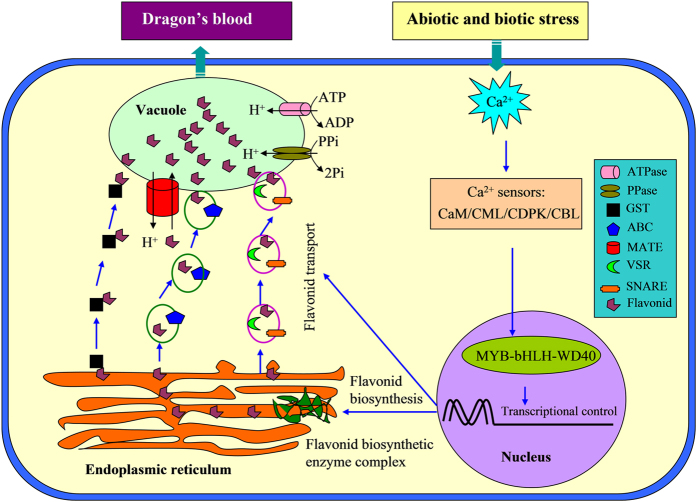
Proposed mechanism of flavonoids accumulation.

The molecular mechanisms of flavonoid biosynthesis, transport and regulation have been well characterized genetically and biochemically in model plants. Many genes involved in the flavonoid biosynthetic pathway have been cloned and characterized. However, the mechanisms of flavonoid biosynthesis and accumulation in *D. cambodiana* are still unknown. In this work, we performed transciptome analysis of three samples with different flavonoid accumulation to identify putative genes for flavonoid metabolism in *D. cambodiana.* As a kind of plant secondary metabolite, the formation of dragon’s blood was considered as a result of plant defense response to biotic and abiotic responses^4^,^5^. Calcium is a critical second messenger in the signal transduction pathways of biotic and abiotic stimuli. Calmodulin (CaM), CaM-like proteins (CMLs), calcium-dependent protein kinases (CDPKs) and calcineurin B-like proteins (CBLs) are major Ca^2+^ sensors, playing critical roles in Ca^2+^-mediated signaling in plant responses to environmental stresses by binding and regulating downstream effectors^47^,^48^. In this study, some Ca^2+^ sensors (7 CDPKs, 14 CaM/CMLs and 1CBL) significantly up-regulated were found from the DEGs (Additional file 5), indicating that Ca^2+^-mediated signaling may be involved in the formation of dragon’s bloodinduced by the inducer. Increasing evidence indicates that calcium plays an import role in flavonoid biosynthesis. It has been reported that the calcium can regulate anthocyanin accumulation, possibly by activating flavonoid pathway genes^49^,^50^,^51^. Several components of calcium signaling pathway such as Ca^2+^, calmodulin, and protein kinases have been shown to be involved in regulating the anthocyanin level^51^,^52^,^53^. Recent research suggests that calcium boost anthocyanin accumulation, possibly by acting on a regulatory gene(s) rather than directly activating structural genes^53^. Based on these previous studies and our transciptome analysis, we proposed a model to explain the mechanism of flavonoid accumulation in *D. cambodiana* (Fig. 7). In this model, different biotic and abiotic stimuli induce transient fluctuations in cytosolic Ca^2+^ levels in *D. cambodiana* and cause the calcium signaling, which is decoded by an array of Ca^2+^ sensors such as CaM/CMLs, CDPKs and CBLs. These sensors interact with transcriptional activators, resulting in transcriptional activation of genes of flavonoid biosynthesis and transport leading to an increase in flavonoid accumulation. The transport of flavonoids from cytosolic synthesis to vacuolar accumulation may occur through three basic mechanisms: membrane transporter-, GST-, or vesicle trafficking-mediated transport. After reprocessed, the secondary metabolites (the main chemical compositions of dragon’s blood) will send out to extracellular in response to a variety of stress. Future studies will focus on verifying this proposed model by identifying putative genes involved in flavonoid accumulation.

In summary, *De novo* characterization of transcriptome of *D. cambodiana* was firstly identified and genes expression was analyzed during formation of dragon’s blood on transcriptome level. A total of 198,204 unigenes were identified from the three cDNA libraries, which will contribute significantly to further research of this specie and other related species. Many candidate genes involved in flavonoid biosynthesis, modification, transport and regulation were identified, which are worthy of further functional research. The results provide insights on pathways and molecular regulation of flavonoid biosynthesis and transport, and improve our understanding of molecular mechanisms of dragon’s blood formation.

## Methods

### Plant materials

Three-year old *Dracaena cambodiana* Pierre ex Gagnep were planted in Hainan Province of China. The stems were injected with the inducter (37.5 g/L NaCl and 1.25 ml/L acetic acid), and samples were taken from 6 cm above the inject site at 3 days and 6 days after transfusing. The stems cut from healthy trees were used to generate material for the 0 day library. All samples were collected, immediately frozen in liquid nitrogen and stored at −80 °C prior to RNA extraction.

### RNA extraction

Total RNAs from stems of three replicates were extracted using the RNA easy plant Mini Kit (QIAGEN) and treated with DNase I (Thermo) according to manufacturer’s instructions. RNA quality was examined using 1% agarose gel and the concentration was determined using Nanodrap (Thermo).

### HPLC analysis

The dried stems of *D. cambodiana* were refluxed with 95% EtOH. The solvent was evaporated under reduced pressure to yield the 95% EtOH extract. The flavonoids in the extract was chromatographed and detected with an Agilent 1260 Infinity HPLC system equipped with a diode array detector (DAD, G4212B) and a C18 Analytical HPLC Column (4.6 × 100 mm, 3.5 um, Agilent) as the stationary phase. The mobile phase consisted of acetonitrile (A) and 0.5% (v/v) formic acid aqueous solution (B), with a gradient elution program, 0–40 min: 10% to 25% A; 40 to 110 min: 25–45% A at a flow rate of 1.0 mL∙min^−1^. The HPLC chromatogram was monitored at 279 nm and the column temperature was set at 30 °C. The compounds were determined by comparing to standards isolated in our previous work and their structures were identified by ^1^HNMR, ^13^CNMR and MS.

### cDNA library construction and Illumina sequencing

Paired-end Illumina mRNA libraries were generated from 4 mg of total RNA following the manufacturer’s instructions for mRNASeq sample preparation (Illumina Inc., San Diego, CA). Library quality was assessed with the 2100 Bioanalyzer (Agilent Technologies, Palo Alto, CA). The samples for transcriptome analysis were prepared using a Truseq^TM^ RNA sample prep kit (Illumina) according to the manufacturer’s recommendations. Briefly, mRNA was isolated from 0.5 mg of total RNA using oligo (dT) magnetic beads. mRNA was cut into short fragments by adding fragmentation buffer. First-strand cDNA was synthesized using random hexamer-primers, taking these short fragments as templates. RNaseH, buffer, dNTPs, and DNA polymerase I were used to synthesize second-strand cDNA (NEBNext^®^ mRNA library prep master mix set for Illumina®). Short fragments were purified with Takara’s PCR extraction kit (Takara Bio, Inc.). Sequencing adapters were ligated to short fragments and resolved by agarose gel electrophoresis. Proper fragments were selected and purified and subsequently PCR amplified to create the final cDNA library template.

### Analysis of transcriptome assembly

The transcriptome was sequenced using the Illumina HiSeq^TM^ 2000. Four fluorescently labeled nucleotides and a specialized polymerase were used to determine the clusters base by base in parallel. The size of the library was approximately 100 bp and both ends of the library were sequenced. The 100 bp raw pairedend reads were generated on the Illumina sequencing platform. Image deconvolution and quality value calculations were performed using Illumina GA pipeline v1.6. The raw reads were cleaned by removing adaptor sequences, empty reads, and low quality sequences (reads with unknown sequences ‘N’ or less than 25 bp). The clean reads were assembled into non-redundant transcripts using the Trinity, which has been developed specifically for the de novo assembly of transcriptomes using short reads.

### Annotation of unigenes

Unigenes were used as query sequences to search against the non-redundant protein (NR) database at NCBI (http:// www.ncbi.nlm.nih.gov) and the Swiss-Prot protein database (http://www.ebi.ac.uk/uniprot) with *E*-value cut-off of 1e-5. The annotations of the best hits were recorded. Gene Ontology (GO) (http://www.geneontology.org/) were further used to category the function of the unigenes by Blast2GO, and the unigenes were assigned to biological functions on the macro levels of “biological process”, “cellular component” and “molecular function”. The Kyoto Encyclopedia of Genes and Genome (KEGG) pathways database (http://www.genome.jp/kegg/) were assigned to unigenes by KEGG Automatic Annotation Server (KAAS).

### Differential expression analysis

Gene expression levels of unigenes in stems were normalized and calculated as fragment per kilobase of exon model per million mapped reads (RPKM) values during the assembly and clustering process. False discovery rate (FDR) < 0.001 and an estimated absolute log2 fold-change (log2 FC) ≥ 1 were used as threshold for identifying differentially expressed genes (DEGs).

### Quantitative real-time RT-PCR

To verify the RNA-Seq results, quantitative RT-PCR was conducted using SYBR-green (TaKaRa Biotechnology Co., Ltd, Dalian, China) and Stratagene Mx3005 P Real Time Thermal Cycler (Agilent, America) with the following program: 95 °C for 30 s, followed by 40 cycles of 95 °C for 10 s, and then annealing at 65 °C–95 °C for 30 s. We used the Primer Premier v. 5.0 and Vector NIT v.11.0 software (Applied Biosystems) to design primers based on the sequences of key genes of interest identified in our library. The *D. cambodiana* actin gene was used as an internal control. The sequences of primers used in this study are provided in Table S6.

## Additional Information

**How to cite this article**: Zhu, J.-H. *et al*. *De Novo* transcriptome characterization of *Dracaena cambodiana* and analysis of genes involved in flavonoid accumulation during formation of dragon’s blood. *Sci. Rep.* **6**, 38315; doi: 10.1038/srep38315 (2016).

**Publisher's note:** Springer Nature remains neutral with regard to jurisdictional claims in published maps and institutional affiliations.

## Supplementary Material

Supplementary Information

Supplementary Dataset 1

## Figures and Tables

**Table 1:** Statistical summary of trimmed Illumina sequencing data, *de novo* assembly and annotation.

Trimmed Illumina sequencing data					
		Number of reads	Total bases	N(%)	GC(%)
Raw	0d	87.09 M	8.62 G	0	47.69
	3d	75.87 M	7.51 G	0	48.03
	6d	103.61 M	10.26 G	0	48.64
Filter	0d	71.49 M	7.08 G	0	47.49
	3d	63.13 M	6.25 G	0	47.91
	6d	82.88 M	8.21 G	0	47.91
*De novo* assembly and annotation		*De novo* assembly			Annotation
Total base(MB)		93.63			34.14
Number of Transcript		198204			34783
Max Unigene length (bp)		21883			21883
Mean Unigene length		495			1029
N50		562			1694
