# Supplementary material for: De Novo transcriptome characterization of Dracaena cambodiana and analysis of genes involved in flavonoid accumulation during formation of dragon’s blood
Source: Sci Rep. 2016 Dec 6;6:38315. doi: 10.1038/srep38315 (PMC5138819; doi:10.1038/srep38315)
Supplement: Supplementary Information [file srep38315-s1.pdf]

Supporting Information

De Novo transcriptome characterization of *Dracaena cambodiana* and analysis of genes involved in flavonoid accumulation during formation of dragon's blood

Jia-Hong Zhu*, Tian-Jun Cao*, Hao-Fu Dai, Hui-Liang Li, Dong Guo, Wen-Li Mei & Shi-Qing Peng

Key Laboratory of Biology and Genetic Resources of Tropical Crops, Ministry of Agriculture, Institute of Tropical Bioscience and Biotechnology, Chinese Academy of Tropical Agricultural Sciences, No.4 Xueyuan Road, Haikou 571101, China.

*These authors contributed equally to this work. Correspondence and requests for materials should be addressed to W.L.M. (email: meiwenli@itbb.org.cn) and S.Q.P. (email: shqpeng@163.com)

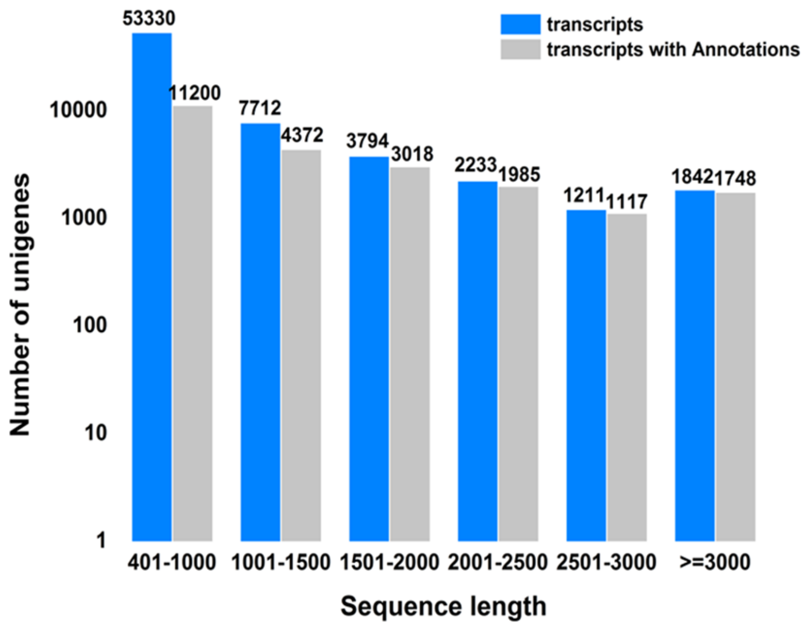

Figure. S1. Length distribution of total unigenes and annotated unigenes longer than 400 bps.

Table S1. Compounds identification of HPLC

Peak no.	t_R(min)[a]	Compounds	t_R(min)[b]
1	29.8581	(*2S*)7,4'-dihydroxyflavanone	29.435
2	41.7282	4,4′ -dihydroxy-2-methoxydihydrochalcone	41.216
3	51.407	(*2S*)-7,3'-dihydroxy-4'-methoxyflavane	51.504
4	56.1551	(*2R*)-7,4'-dihydroxy-8-methylflavane	55.517
5	61.5437	(*2S*)-3',7-dihydroxy-4'-methoxy-8-methylflavane	61.607
6	69.0297	(*3R*)-3,5,7-trihydroxy-4'-methoxy-6-methoxydihydro-homoisoflavone	68.978

a：retention time of tests   b: retention time of controls

Table S3. GO terms enriched in 3d/0d and 6d/0d

GO_ID	GO_Term	GO_Class	Pvalue	Corrected_P-value	Unigene_diff	Unigene_diff_all
3d vs 0d						
GO:0009055	electron carrier activity	molecular_function	1.97E-08	6.53E-07	56	1296
GO:0005576	extracellular region	cellular_component	3.18E-08	6.53E-07	31	1296
GO:0001071	nucleic acid binding transcription factor activity	molecular_function	3.18E-06	4.34E-05	59	1296
GO:0005215	transporter activity	molecular_function	1.97E-05	2.02E-04	116	1296
GO:0016020	membrane	cellular_component	7.24E-05	5.94E-04	145	1296
GO:0044425	membrane part	cellular_component	1.38E-03	0.009425919	169	1296
6d vs 0d						
GO:0005215	transporter activity	molecular_function	2.90E-10	1.22E-08	168	1710
GO:0003824	catalytic activity	molecular_function	1.72E-07	3.61E-06	1107	1710
GO:0044425	membrane part	cellular_component	1.58E-06	2.22E-05	238	1710
GO:0005576	extracellular region	cellular_component	3.26E-05	3.42E-04	30	1710
GO:0009055	electron carrier activity	molecular_function	0.000183	0.001534471	55	1710
GO:0044699	single-organism process	biological_process	0.000371	0.002598757	546	1710
GO:0001071	nucleic acid binding transcription factor activity	molecular_function	0.00121	0.007260658	62	1710
GO:0016209	antioxidant activity	molecular_function	0.002799	0.014695718	24	1710
GO:0030234	enzyme regulator activity	molecular_function	0.006225	0.029047798	19	1710

Table S4. KEGG pathways enriched in 0d/3d and 0d/6d

KEGG_pathway	ko_id	P-value	Corrected_P-value	gene_diff	gene_diff_all
3d vs 0d					
Phenylpropanoid biosynthesis	ko00940	0	0	49	200
Flavonoid biosynthesis	ko00941	1.444E-11	2.203E-09	25	67
Stilbenoid, diarylheptanoid and gingerol biosynthesis	ko00945	3.511E-08	3.57E-06	25	93
Pentose and glucuronate interconversions	ko00040	2.329E-07	1.433E-05	36	182
Plant-pathogen interaction	ko04626	2.35E-07	1.433E-05	75	521
Phenylalanine metabolism	ko00360	4.623E-07	2.35E-05	30	141
Biosynthesis of secondary metabolites	ko01110	1.002E-06	4.365E-05	245	2356
Polycyclic aromatic hydrocarbon degradation	ko00624	1.441E-05	0.0005495	22	103
Isoquinoline alkaloid biosynthesis	ko00950	1.983E-05	0.000672	11	32
Flavone and flavonol biosynthesis	ko00944	3.996E-05	0.0012188	7	14
Plant hormone signal transduction	ko04075	4.765E-05	0.0013212	69	541
Photosynthesis - antenna proteins	ko00196	8.807E-05	0.0022385	10	31
Drug metabolism - cytochrome P450	ko00982	0.0001872	0.004393	15	67
Bisphenol degradation	ko00363	0.0002148	0.0045331	20	106
Metabolism of xenobiotics by cytochrome P450	ko00980	0.0002229	0.0045331	15	68
Chemical carcinogenesis	ko05204	0.0002734	0.0052118	12	48
Glucosinolate biosynthesis	ko00966	0.0002965	0.0053189	5	9
Carbon fixation in photosynthetic organisms	ko00710	0.000361	0.0061164	23	135
Starch and sucrose metabolism	ko00500	0.0003977	0.0061835	77	664
Cyanoamino acid metabolism	ko00460	0.0004055	0.0061835	20	111
Metabolic pathways	ko01100	0.0006812	0.0098665	391	4344
Aminobenzoate degradation	ko00627	0.0007117	0.0098665	25	159

alpha-Linolenic acid metabolism	ko00592	0.0007861	0.0104244	15	76
Limonene and pinene degradation	ko00903	0.001335	0.0169653	19	113
Tryptophan metabolism	ko00380	0.0016513	0.0201455	19	115
Photosynthesis	ko00195	0.004248	0.047752	21	143
Betalain biosynthesis	ko00965	0.0043838	0.047752	3	5
Steroid degradation	ko00984	0.0043838	0.047752	3	5

6d vs 0d

#KEGG_pathway	ko_id	P-value	Corrected_P-value	gene_diff	gene_diff_all
Metabolic pathways	ko01100	0	0	612	4344
Biosynthesis of secondary metabolites	ko01110	3.484E-11	5.505E-09	360	2356
Flavonoid biosynthesis	ko00941	1.575E-10	1.659E-08	28	67
Phenylpropanoid biosynthesis	ko00940	4.158E-09	3.285E-07	52	200
Photosynthesis - antenna proteins	ko00196	2.864E-07	1.81E-05	15	31
Microbial metabolism in diverse environments	ko01120	2.145E-05	0.0011295	166	1093
Flavone and flavonol biosynthesis	ko00944	4.07E-05	0.0018375	8	14
Diterpenoid biosynthesis	ko00904	8.432E-05	0.0033307	13	36
Stilbenoid, diarylheptanoid and gingerol biosynthesis	ko00945	0.0001962	0.0068905	23	93
Pentose and glucuronate interconversions	ko00040	0.000245	0.0077423	37	182
Phenylalanine metabolism	ko00360	0.0004108	0.0118016	30	141
Steroid degradation	ko00984	0.0007286	0.0191863	4	5
Brassinosteroid biosynthesis	ko00905	0.0014741	0.0358327	11	36

Table S5. A summary of putative unigenes involved in flavonoid accumulation

Gene ID	**Functional annotation**	**RPKM**		
		0 d	3 d	6 d
**Structural genes**				
comp90646_c0_seq1	Phenylalanine ammonia lyase	62.2835	78.1548	108.3744
comp51778_c1_seq1	Phenylalanine ammonia lyase	1.7028	0.6358	0.701
comp2089722_c0_seq1	Phenylalanine ammonia lyase	0.5194	0.0738	0.2238
comp48278_c0_seq1	Phenylalanine ammonia lyase	1.5049	0.2281	0.2882
comp104046_c0_seq3	Phenylalanine ammonia lyase	26.7196	13.4504	18.6697
comp93964_c0_seq2	Phenylalanine ammonia lyase	3.1068	2.1198	1.4282
comp95964_c0_seq1	cinnamate 4-hydroxylase	131.046	165.1452	226.4042
comp102027_c0_seq5	4-coumarate-CoA Ligase	49.2147	10.9281	15.2015
comp100007_c2_seq1	4-coumarate-CoA Ligase	55.5161	208.0091	**225.7814**
comp68498_c0_seq1	4-coumarate-CoA Ligase	4.6314	49.0806	**61.1977**
comp43132_c0_seq1	4-coumarate-CoA Ligase	14.4101	29.3181	**23.5007**
comp86431_c0_seq2	4-coumarate-CoA Ligase	8.5738	**17.7434**	17.5983
comp102113_c0_seq1	4-coumarate-CoA Ligase	7.2751	4.1662	3.8397
comp102535_c0_seq2	4-coumarate-CoA Ligase	5.6051	4.5956	3.7294
comp95083_c0_seq2	4-coumarate-CoA Ligase	1.9066	**4.6689**	3.2264
comp102288_c1_seq1	4-coumarate-CoA Ligase	15.1921	16.9682	13.8495
comp97070_c0_seq2	4-coumarate-CoA Ligase	0.4661	0.7289	0.5901
comp79835_c0_seq2	4-coumarate-CoA Ligase	0.5558	1.1622	0.7573
comp79310_c0_seq1	4-coumarate-CoA Ligase	6.0965	3.3962	6.0363
comp98128_c0_seq1	4-coumarate-CoA Ligase	1.6228	1.9293	1.2716
comp57069_c0_seq1	4-coumarate-CoA Ligase	0.3159	0.6468	0.9261

comp44159_c0_seq1,	4-coumarate-CoA Ligase	3.4709	1.779	2.9497
comp65044_c1_seq1	4-coumarate-CoA Ligase	1.5643	0.2223	0.5056
comp44364_c0_seq1	4-coumarate-CoA Ligase	2.2297	1.6135	1.7472
comp44364_c1_seq1	4-coumarate-CoA Ligase	3.6166	3.5878	3.2502
comp98110_c0_seq1	chalcone synthase	2.9353	34.5344	**37.4429**
comp81446_c0_seq1	chalcone synthase	0.2672	2.5526	**3.4551**
comp58784_c0_seq1	chalcone synthase	2.2769	1.1651	1.3983
comp98671_c0_seq1	chalcone synthase	0.5791	0.922	**1.2979**
comp38055_c0_seq1	chalcone synthase	0.2829	0.4826	**1.3717**
comp3620420_c0_seq1	chalcone synthase	0.3259	0	0.4013
comp45413_c0_seq1	chalcone synthase	0	2.6901	**4.8294**
comp106286_c2_seq2	chalcone synthase	1.1268	**26.2103**	17.3477
comp5780917_c0_seq1	chalcone synthase	0.3061	0.24367	0
comp7813316_c0_seq1	chalcone synthase	0.2027	0.2881	0
comp88411_c0_seq1	chalcone isomerase	0.61269	0.6252	0.4062
comp55258_c1_seq1	chalcone isomerase	0.201	0.1524	0.1733
comp116889_c0_seq1	chalcone isomerase	3.2999	13.8368	**51.4985**
comp55258_c0_seq1	chalcone isomerase	0.4456	0.2027	0.6145
comp117347_c0_seq1	chalcone isomerase	0.3015	15.2434	**22.3433**
comp4668674_c0_seq1	chalcone isomerase	0.2149	0.4073	0
comp98940_c0_seq2	Dihydroflavonol 4-reductase	98.6971	59.0352	90.9577
comp89340_c0_seq2	Dihydroflavonol 4-reductase	2.6654	2.719	2.4595
comp43154_c0_seq1	Dihydroflavonol 4-reductase	7.4693	10.3491	**15.9037**
comp68713_c0_seq4	Dihydroflavonol 4-reductase	7.6225	5.8664	6.2801
comp94880_c0_seq1	Dihydroflavonol 4-reductase	0.8803	4.644	**13.8154**
comp85451_c0_seq1,	Dihydroflavonol 4-reductase	1.0224	15.9	**14.7476**

comp89845_c0_seq1,	Dihydroflavonol 4-reductase	2.32586	6.8866	**5.1833**
comp93538_c0_seq1	Dihydroflavonol 4-reductase	7.5073	6.0957	4.9227
comp90404_c0_seq1	Dihydroflavonol 4-reductase	4.2962	2.277	2.7314
comp94270_c0_seq1	Dihydroflavonol 4-reductase	5.9566	4.95825	3.1054
comp75694_c0_seq2	Dihydroflavonol 4-reductase	1.0687	0.7995	**1.8909**
comp103695_c0_seq1	Dihydroflavonol 4-reductase	11.5925	20.31303	**28.5986**
comp1808121_c0_seq1	Dihydroflavonol 4-reductase	0	0.2271	0.4132
comp178014_c0_seq1	Dihydroflavonol 4-reductase	0.6868	**3.5929**	2.932
comp104582_c0_seq1	Dihydroflavonol 4-reductase	205.1457	106.1608	106.1608
comp2789080_c0_seq1	Dihydroflavonol 4-reductase	0	0.1321	0.5009
comp101612_c0_seq2	leucoanthocyanidin reductase	7.4994	26.6989	**43.4428**
comp100166_c0_seq1	flavanone 3-hydroxylase	0	5.183	**13.7723**
comp56219_c0_seq1	flavanone 3-hydroxylase	0.5631	0.661	0.548
comp260931_c0_seq1	flavanone 3-hydroxylase	0.2757	**1.7617**	1.7315
comp662391_c0_seq1	flavanone 3-hydroxylase	0.7993	0.909	0.2952
comp245016_c0_seq1	flavanone 3-hydroxylase	1.9264	1.1208	1.0426
comp4887219_c0_seq1	flavanone 3-hydroxylase	0.2643	0.1503	0.1424
comp7848127_c0_seq1	flavanone 3-hydroxylase	0.13967	0	0.1605
comp67685_c0_seq2	Flavonol synthase	4.218	3.6332	4.418
comp43824_c0_seq1	Flavonol synthase	206.4677	238.9395	144.9218
comp99303_c0_seq1	Flavonol synthase	0.438	1.5347	**8.2556**
comp37699_c0_seq1	Flavonol synthase	0.1304	1.4468	**1.4903**
comp107388_c1_seq1	Flavonol synthase	195.9822	61.404	61.6016
comp662391_c0_seq1	Flavonol synthase	0.7993	0.909	0.2952
comp43164_c0_seq1	Flavonol synthase	47.3195	49.8714	44.3824
comp104393_c0_seq1	Flavonol synthase	29.9423	41.4778	40.5916

comp4887219_c0_seq1	Flavonol synthase	0.2643	0.1503	0.1424
comp7848127_c0_seq1	Flavonol synthase	0.13967	0	0.16053
**Modification genes**				
comp85963_c0_seq1	Cytochrome P450 71A1	4.550641753	19.16502298	15.60728843
comp105256_c0_seq4	Cytochrome P450 71A9	0	2.177636827	12.02600945
comp106175_c0_seq1	Cytochrome P450 71A9	41.68295101	8.000187155	27.9457139
comp99358_c0_seq3	Cytochrome P450 72A154	0.377521026	58.54218706	36.88077398
comp101104_c0_seq4	Cytochrome P450 734A6	0.754006674	5.913932828	3.373977659
comp43483_c0_seq1	Cytochrome P450 74A1	1.479650904	18.16151698	34.29236573
comp112145_c0_seq1	Cytochrome P450 71D55	0.056503383	34.62311773	38.61513627
comp99725_c0_seq1	Cytochrome P450 81E1	0.457561307	57.71240623	66.64711397
comp101897_c2_seq2	Cytochrome P450 85A1	3.076849072	46.47361165	63.31365117
comp76767_c0_seq1	Cytochrome P450 86B1	0.441631715	4.337389679	4.741211794
comp71602_c1_seq2	Cytochrome P450 90B1	0.231185105	2.021640742	6.926655731
comp43439_c0_seq1	UDP-glucose flavonoid 3-O-glucosyl transferase	28.48922922	122.3658194	77.19823668
comp81737_c0_seq1	UDP-glucose flavonoid 3-O-glucosyl transferase	0	0.508985959	2.683110163
comp95164_c0_seq1	UDP-glucose flavonoid 3-O-glucosyl transferase	0	1.189332257	1.792273714
comp97509_c1_seq1	UDP-glucose flavonoid 3-O-glucosyl transferase	0.172540607	15.06482626	17.68209869
comp97909_c0_seq1	UDP-glucose flavonoid 3-O-glucosyl transferase	0.035477933	10.81260596	15.48955655
comp97393_c0_seq1	UDP-glucose flavonoid 3-O-glucosyl transferase	0.935368295	42.72692633	97.55229057
comp103204_c0_seq1	UDP-glucose flavonoid 3-O-glucosyl transferase	0.737347505	30.06521521	36.17472443
comp68451_c1_seq1	Anthocyanidin 3-O-glucoside 5-O-glucosyltransferase	0.800817486	141.4909503	116.0079204
comp97909_c0_seq1	Anthocyanidin 3-O-glucoside 5-O-glucosyltransferase	0.035477933	10.81260596	15.48955655
comp103204_c0_seq1	Anthocyanidin 3-O-glucoside 5-O-glucosyltransferase	0.737347505	30.06521521	36.17472443
comp105806_c0_seq2	Anthocyanidin 3-O-glucoside 5-O-glucosyltransferase	16.38691888	43.34100879	53.43368811
comp99832_c1_seq1	Isoflavone 7-O-glucosyltransferase	1.806370788	18.0579112	17.92440981

comp101724_c0_seq1	Isoflavone 7-O-glucosyltransferase	48.66684439	152.5998549	199.9021456
comp105806_c0_seq2	Isoflavone 7-O-glucosyltransferase	16.38691888	43.34100879	53.43368811
comp87786_c0_seq1	O-methyltransferase (OMT)	0.058320743	0.994836192	4.423916713
comp103635_c0_seq1	O-methyltransferase (OMT)	7.769772392	56.70566296	75.85734093
**Transporter-related genes**				
comp99981_c0_seq1	Multidrug and toxic compound extrusion protein (MATE)	2.6578	22.0301	32.3219
comp82304_c0_seq1	Multidrug and toxic compound extrusion protein (MATE)	0.8629	1.7757	1.4433
comp36251_c0_seq1	Multidrug and toxic compound extrusion protein (MATE)	0.5802	0.9703	2.3388
comp99977_c0_seq1	Multidrug and toxic compound extrusion protein (MATE)	0.8621	1.938	2.2122
comp97344_c0_seq1	Multidrug and toxic compound extrusion protein (MATE)	2.3924	4.797	4.385
comp87015_c0_seq1	Multidrug and toxic compound extrusion protein (MATE)	0.2423	1.8373	0.922
comp53053_c0_seq1,	Multidrug and toxic compound extrusion protein (MATE)	0.0234	2.289	0.8273
comp83276_c0_seq1	Multidrug and toxic compound extrusion protein (MATE)	0.7183	1.6338	2.2847
comp111080_c0_seq1	Multidrug and toxic compound extrusion protein (MATE)	3.4341	47.5515	48.5002
comp103423_c1_seq3	Multidrug and toxic compound extrusion protein (MATE)	4.1328	16.5379	16.7541
comp105283_c0_seq5	Multidrug and toxic compound extrusion protein (MATE)	0.4947	3.4111	6.4173
comp114619_c0_seq1	Multidrug and toxic compound extrusion protein (MATE)	0.0777	15.0021	17.8025
comp36366_c0_seq1	Multidrug and toxic compound extrusion protein (MATE)	0.4104	2.1391	1.474
comp105224_c2_seq1	Multidrug and toxic compound extrusion protein (MATE)	12.1865	25.2696	15.3554
comp37394_c0_seq1	Multidrug and toxic compound extrusion protein (MATE)	0.9777	1.8234	2.3261
comp545163_c0_seq1	Multidrug and toxic compound extrusion protein (MATE)	0.4134	0.6112	1.782
comp103580_c0_seq9	Multidrug and toxic compound extrusion protein (MATE)	0.13593	3.2805	2.109
comp306531_c0_seq1	Multidrug and toxic compound extrusion protein (MATE)	0.4571	1.5968	0.8022
comp98856_c0_seq6	ABC transporter C	2.44	6.7266	10.8537
comp45417_c0_seq1	ABC transporter C	3.3121	16.7421	20.1335
comp42540_c0_seq1	ABC transporter C	2.2396	4.73817	7.4766

comp70236_c0_seq1	ABC transporter C	0.63473	1.8351	3.1529
comp103464_c2_seq1	ABC transporter C	1.2552	3.6414	7.2147
comp45417_c1_seq1	ABC transporter C	0.6698	1.7854	2.4721
comp46591_c0_seq1	ABC transporter C	0.6809	2.1823	3.7352
comp151322_c0_seq1	ABC transporter C	2.0357	5.0115	9.761
comp207602_c0_seq1	ABC transporter C	0.2201	1.2517	4.7125
comp87983_c0_seq1	ABC transporter C	0.0336	0.5739	1.7691
comp87190_c0_seq2	ABC transporter C	0.2098	0.8353	1.2844
comp40727_c0_seq1	ABC transporter C	0	0.7523	1.9602
comp427607_c0_seq1	ABC transporter C	0	0.6016	1.406
comp86364_c0_seq2	ABC transporter G	0.1758	3.2816	2.0951
comp102744_c1_seq2	ABC transporte G	0.6235	3.8114	3.191
comp41925_c0_seq1	ABC transporter G	0	0.825	1.8193
comp102946_c0_seq1	Glutathione S-transferase	2.3575	12.7105	11.2134
comp42858_c0_seq1	Glutathione S-transferase	1.0517	4.2352	3.7899
comp44529_c0_seq1	Glutathione S-transferase	0.0177	4.7204	5.9328
comp96056_c0_seq1	Glutathione S-transferase	35.4446	335.1131	606.0485
comp121414_c0_seq1	Glutathione S-transferase	0	35.6836	13.425
comp93461_c0_seq1	Glutathione S-transferase	0.3291	3.4154	0.9043
comp92860_c0_seq1	Glutathione S-transferase	0.1761	180.0561	191.3806
comp113922_c0_seq1	Glutathione S-transferase	12.1787	45.254	40.1638
comp102042_c0_seq4	vacuolar sorting receptor	22.9555	40.9078	49.0877
comp105510_c1_seq1	vacuolar sorting receptor	34.8862	228.7368	251.6733
comp97466_c0_seq1	N-ethylmaleimidesensitive factor attachment protein receptors (SNARE)	2.4984	6.2743	6.0919
comp102875_c0_seq2	N-ethylmaleimidesensitive factor attachment protein receptors (SNARE)	1.3203	2.8027	2.782
comp474487_c0_seq1	N-ethylmaleimidesensitive factor attachment protein receptors (SNARE)	0.2123	1.5521	0.5751

comp91790_c0_seq1	N-ethylmaleimidesensitive factor attachment protein receptors (SNARE)	6.3723	14.5289	11.1075
comp176479_c0_seq1	H+-ATPase	1.3801	6.2778	7.1378
comp96852_c0_seq1	H+-ATPase	18.4244	41.1539	36.6466
comp100242_c0_seq1	H+-ATPase	11.4955	26.0367	24.26
comp99491_c0_seq3	H+-ATPase	16.3327	60.7486	53.3154
comp85998_c0_seq1	H+-ATPase	5.72471	18.812	15.1782
comp95829_c0_seq1	H+-ATPase	5.4193	17.4363	17.0761
comp106124_c1_seq2	H+-ATPase	5.1434	93.9068	86.6855
comp67154_c0_seq1	H+-ATPase	7.75985	17.1315	13.6461
comp103331_c0_seq1	H+-ATPase	3.0554	15.462	11.2986
comp139666_c0_seq1	H+-ATPase	1.8293	15.7455	18.81328
comp109604_c0_seq1	H+-ATPase	10.67457	76.126	116.0976
comp87097_c0_seq1	H+-ATPase	1.8386	5.6036	3.80374
comp189990_c0_seq1	H+-ATPase	0.17682	5.12763	8.76413
comp62923_c0_seq1	H+-ATPase	3.7589	7.4138	7.6446
comp103887_c1_seq1	H+-ATPase	47.3884	123.5231	123.5231
comp176479_c0_seq1	H+-ATPase	1.380117	6.27788	7.13786
comp329432_c0_seq1	H+-ATPase	0.67466	2.4412	2.1147
comp155454_c0_seq1	H+-ATPase	1.42646	5.78341	10.9594
comp42896_c0_seq1	H+-pyrophosphatase	0.53834	2.0661	1.2181
comp100999_c0_seq1	H+-pyrophosphatase	0.25637	2.7534	3.17978
**Transcription factors**				
comp98933_c0_seq1	MYB transcription factor	24.30066	1.6330674	2.5020376
comp96546_c1_seq1	MYB transcription factor	7.790301	2.1759288	2.4558766
comp93982_c0_seq7	MYB transcription factor	14.38032	5.176781	5.2218753
comp96293_c0_seq2	MYB transcription factor	2.651041	1.7527714	1.0734964

comp53072_c0_seq	MYB transcription factor	5.742902	2.3871327	2.7312071
comp100596_c0_seq2	MYB transcription factor	7.238577	1.1338026	1.918961
comp89126_c0_seq1	MYB transcription factor	85.55917	25.936622	33.698948
comp98372_c0_seq3	MYB transcription factor	4.147972	0.2685881	0.6743829
comp79556_c0_seq1	MYB transcription factor	9.129861	2.0832725	2.1568215
comp76183_c0_seq2	MYB transcription factor	6.704435	2.947353	3.839384
comp46155_c0_seq2	MYB transcription factor	9.596048	0.9210665	1.2749008
comp91724_c1_seq2	MYB transcription factor	2.030096	0.2885784	0.4218553
comp59683_c0_seq1	MYB transcription factor	14.98031	3.6368188	5.4226706
comp119286_c0_seq1	MYB transcription factor	19.64348	10.233179	8.2037816
comp44654_c0_seq1	MYB transcription factor	47.25576	30.180933	18.301496
comp94385_c0_seq3	MYB transcription factor	3.17809	0.9396731	0.7670531
comp43712_c0_seq1	MYB transcription factor	13.46259	5.5166142	5.5753907
comp104801_c0_seq19	MYB transcription factor	14.60922	4.9996313	4.941631
comp217820_c0_seq1	MYB transcription factor	5.45699	0.8094384	1.2725418
comp106263_c2_seq10	MYB transcription factor	28.26861	10.846173	8.9869623
comp41368_c0_seq1	MYB transcription factor	3.915621	0.1817489	0.482174
comp42650_c0_seq1	MYB transcription factor	3.729682	2.3051082	1.8171409
comp283535_c0_seq1	MYB transcription factor	2.958503	0.9787385	0.9273435
comp102716_c0_seq1	MYB transcription factor	15.97867	36.483393	37.585001
comp69349_c0_seq2	MYB transcription factor	5.118806	21.611715	14.648663
comp103150_c1_seq5	MYB transcription factor	0.911723	5.8107068	4.3181005
comp102395_c0_seq1	MYB transcription factor	1.314359	3.8888418	3.7498034
comp101464_c0_seq1	MYB transcription factor	8.129308	57.897536	54.275235
comp100498_c0_seq1	MYB transcription factor	0.041484	5.0163911	9.1185565
comp45114_c0_seq1	MYB transcription factor	0.307424	9.928735	13.656134

comp46634_c0_seq1	MYB transcription factor	0.525578	3.3619958	6.3237131
comp98725_c0_seq1	MYB transcription factor	1.208381	2.7203013	6.823611
comp46547_c0_seq1	MYB transcription factor	0.619277	3.9437619	3.3523261
comp89810_c1_seq1	MYB transcription factor	0.049616	2.6236948	3.4856351
comp89810_c0_seq1	MYB transcription factor	0	**1.1938034**	1.0180035
comp36745_c0_seq2	MYB transcription factor	0.484118	2.2633328	1.7155855
comp42816_c0_seq2	MYB transcription factor	1.315594	4.196365	5.0063123
comp93902_c2_seq1	MYB transcription factor	0.255153	1.4508027	3.1419863
comp46263_c0_seq1	MYB transcription factor	0.874811	4.3109568	6.499423
comp88647_c1_seq2	MYB transcription factor	0.237448	3.1863182	3.111105
comp61623_c0_seq1	MYB transcription factor	0.356901	1.7394375	2.1535135
comp105885_c1_seq1	MYB transcription factor	2.960523	59.815717	53.624972
comp105611_c2_seq3	bHLH transcription factor	5.698299	2.401949	2.5233644
comp43888_c0_seq1	bHLH transcription factor	42.85837	2.2506989	1.6984604
comp85163_c0_seq2	bHLH transcription factor	7.169125	2.9495253	2.9678304
comp96407_c1_seq10	bHLH transcription factor	45.98843	7.4357482	11.738059
comp97534_c0_seq2	bHLH transcription factor	2.099876	1.3187363	0.7834624
comp94044_c1_seq1	bHLH transcription factor	8.971784	2.9593864	3.6180448
comp57888_c0_seq1	bHLH transcription factor	17.52499	9.679288	6.404085
comp78551_c0_seq2	bHLH transcription factor	19.47045	10.436709	8.9559672
comp93081_c1_seq4	bHLH transcription factor	6.330117	1.3911436	3.0295208
comp45754_c0_seq1	bHLH transcription factor	19.00147	7.8536012	5.2298583
comp88231_c0_seq1	bHLH transcription factor	2.590619	0.7306215	0.6073984
comp85010_c0_seq3	bHLH transcription factor	3.095021	1.1173529	2.7102185
comp97672_c2_seq1	bHLH transcription factor	6.722437	1.3161314	1.0612931
comp64143_c0_seq1	bHLH transcription factor	2.994298	1.1813679	0.6584309

comp79305_c0_seq1	bHLH transcription factor	17.81381	4.6021987	5.3572232
comp63178_c0_seq1	bHLH transcription factor	5.209102	0.6782974	1.1653912
comp90322_c0_seq1	bHLH transcription factor	15.47656	4.5325955	2.7777723
comp125639_c0_seq1	bHLH transcription factor	17.39486	4.326073	3.3743679
comp85864_c0_seq1	bHLH transcription factor	1.19843	4.0188773	6.5444961
comp95502_c0_seq1	bHLH transcription factor	12.41046	25.15247	20.754448
comp85813_c0_seq3	bHLH transcription factor	9.797884	20.311238	21.032456
comp100958_c0_seq1	bHLH transcription factor	5.040677	8.6040046	11.164363
comp92986_c0_seq2	bHLH transcription factor	6.114612	19.893502	13.223204
comp93262_c0_seq3	bHLH transcription factor	5.897185	16.946966	21.520626
comp44431_c0_seq1	bHLH transcription factor	8.47366	17.872373	14.577173
comp99084_c0_seq1	bHLH transcription factor	3.436815	23.744091	9.0841061
comp102813_c0_seq4	bHLH transcription factor	0.333699	7.0475489	6.4651624
comp91270_c1_seq1	bHLH transcription factor	0.36353	2.7330797	1.9846052
comp45679_c0_seq1	bHLH transcription factor	0.832557	3.3813752	3.1269227
comp94938_c1_seq1	bHLH transcription factor	0.536174	2.1848956	2.9656296
comp395339_c0_seq1	bHLH transcription factor	0.826673	0.2800279	2.2893609
comp66961_c0_seq1	bHLH transcription factor	2.566541	41.69534	20.753743
comp101975_c0_seq1	WD40 transcription factor	31.49731	51.224708	70.971068
comp95046_c0_seq1	WD40 transcription factor	2.20889815	2.2135105	5.4010744
comp264508_c0_seq1	WD40 transcription factor	1.0395294	1.1821539	2.1448289
comp99035_c0_seq1	WD40 transcription factor	1.422266	2.8002796	2.0310955
comp95046_c0_seq1	WD40 transcription factor	2.20889815	2.2135105	5.4010744
comp82082_c0_seq2	WD40 transcription factor	3.29466737	2.2732782	1.5954852
comp95612_c0_seq3	WD40 transcription factor	3.30563519	1.3301685	1.0155617
comp95612_c0_seq3	WD40 transcription factor	3.30563519	1.3301685	1.0155617

comp159571_c0_seq1	WD40 transcription factor	2.87678281	0.999619	1.7220501
comp546056_c0_seq1	WD40 transcription factor	2.41907871	0.9761538	1.210771
comp363160_c0_seq1	WD40 transcription factor	2.44337968	0.5849713	1.3024961
comp35222_c0_seq1	WD40 transcription factor	2.00773	0.8234485	0.6525376
**Ca2+ sensors**				
comp100473_c0_seq4	Calcium-dependent protein kinase (CDPK)	15.5775	33.0144	33.05999
comp99400_c0_seq3	Calcium-dependent protein kinase (CDPK)	10.6360524	24.1906	18.8462
comp46925_c0_seq1	Calcium-dependent protein kinase (CDPK)	2.3445	6.59423	4.47507
comp46925_c1_seq1	Calcium-dependent protein kinase (CDPK)	2.33282	6.78529	3.9444
comp49831_c0_seq2	Calcium-dependent protein kinase (CDPK)	5.27519	10.6791	11.87748
comp101022_c0_seq1	Calcium-dependent protein kinase (CDPK)	0.65182	1.01921	2.31767
comp103754_c0_seq3	Calmodulin (CaM)	15.97946	29.1436	33.09257
comp103841_c1_seq1	Calmodulin (CaM)	128.57987	337.2324	305.0934
comp43749_c0_seq1	CaM-like protein (CML)	1.0061	96.1597	84.9806
comp68074_c0_seq1	CaM-like protein (CML)	10.51332	21.25936	16.36967
comp96864_c0_seq1	CaM-like protein (CML)	0.60784	6.745566	8.07613
comp37573_c0_seq1	CaM-like protein (CML)	0.38754	1.15076	3.0065
comp45520_c1_seq1	CaM-like protein (CML)	20.8339	45.5279	40.1393
comp93832_c0_seq1	CaM-like protein (CML)	0.51849	6.33432	8.862
comp96864_c0_seq1	CaM-like protein (CML)	0.60784	6.7455	8.07613
comp101899_c0_seq9	CaM-like protein (CML)	2.52998	5.68729	5.46049
comp110824_c0_seq1	CaM-like protein (CML)	14.2717	38.8329	50.67869
comp172533_c0_seq1	CaM-like protein (CML)	1.89006	10.3991	4.45636
comp268011_c0_seq1	CaM-like protein (CML)	1.61918	4.96033	1.2532
comp70694_c0_seq1	CaM-like protein (CML)	0.38654	9.30441	9.08886
comp91249_c0_seq3	Calcineurin B-like protein (CBL)	4.251	8.725807	6.302615

Table S6. Primers used in validation experiment of gene expression by qRT-PCR

Gene ID	Primer name	sequence（5’- 3’）
comp45413_c0_seq1	q-CHS1-F	TCTACCGATGGAGAAGATGG
	q-CHS1-R	AAGTAGTAGTCGGCGTAAGT
comp98110_c0_seq1	q-CHS2-F	CGTGAACTGACTCCGATTG
	q-CHS2-R	CAATACCCTTCTTCCCACATC
comp106286_c2_seq2	q-CHS3-F	GGACTTGGTTACTTTGCATTTC
	q-CHS3-R	CTCGACACATGTTATAGAACCC
comp81446_c0_seq1	q-CHS4-F	CGGACTACTACTTCCGTATCA
	q-CHS4-R	ATCGACTTGTCACACATTCTC
comp117347_c0_seq1	q-CHI1-F	CACGGTGTATCTCCTATGTTAAA
	q-CHI1-R	GACACACGCATAGTCATCTC
comp116889_c0_seq1	q-CHI2-F	CTTCCCTGCGAGTTCTTATAC
	q-CHI2-R	CTGTAGACCCACCCAAATAC
comp94880_c0_seq1	q-DFR-F	CACAAGCCAAGGGAAGATAC
	q-DFR-R	CCTTCGAACTGAGTAGGGATA
comp101612_c0_seq2	q-LAR-F	GAAGCTGAGAGAGCACCATATT
	q-LAR-R	CTCTGATGGCAGAAACCTCTT
comp96056_c0_seq1	q-GST-F	CGAGGAAGCCAAGAAGGAAT
	q-GST-R	GGTGAAGGGCACAAGAGTAA
comp98856_c0_seq6	q-ABC-F	GGAGACTGTGAGTGCTGTTATT
	q-ABC-R	GACCATAAGACAGAGCCATTCC
comp111080_c0_seq1	q-MATE-F	ACTGTGGTTCAGACGCTAATC
	q-MATE-R	TTCCATTCTCGACGCCTTTC
comp95829_c0_seq1	q-ATPA-F	CCGAGCTTCGTTAGAGAGTAAAT
	q-ATPA-R	CTTCATGTCTGTCTCCTCATCAA
comp100498_c0_seq1	q-MYB-F	TACCGCATGACGTCCTTAAAC
	q-MYB-R	GGGAGGACCGATAACGAAATAAA
comp85864_c0_seq1	q-HLH-F	AAAGATCCACCCTACCTGTTTC

	q-HLH-R	TGGAGTTAGCCTTGGAAGTAAAG
comp101975_c0_seq1	q-WD40-F	CAGCAAGGTCCATACACTACTC
	q-WD40-R	TCTTGCCGTGTCCTGTAATC
	q-ACT-F（actin）	ACCGAGAGAGGGTACTCATT
	q-ACT-R（actin）	CCAGCTCCTGCTCGTAATC
